# Everyday nuclear histories and futures in the Middle East, 1945–1948

**DOI:** 10.1080/09557571.2023.2275611

**Published:** 2023-12-17

**Authors:** Hebatalla Taha

**Affiliations:** Lund University

## Abstract

This article examines nuclear imaginaries in the Arabic-speaking Middle East. It situates people from the Arab world into nuclear thought, looking at how the atomic age rapidly became part of everyday lives. Embracing the idea that reality and fiction are not only deeply intertwined but also co-constitutive, it analyses everyday engagements with the nuclear condition in the aftermath of the bombing of Japan, across a wide range of sources. The article argues that these semi-fictional historical sources—memoirs, pseudo-scientific predictions, speculative reports published in newspapers, popular science books and even rumours—capture an affective moment at the beginning of the atomic age, which was marked by hysteria, widespread speculation and exaggeration. Discussions on nuclear weapons, precisely because the extent of their destruction seems unimaginable, blur the boundaries between ‘real’ and ‘fictional’, offering a unique opportunity to reflect on stories of world politics and on the tensions within historical International Relations.

## Introduction

What I remember of Hiroshima is the American attempt to make it forget its name. I know Hiroshima. I was there nine years ago. In one of its squares, it spoke of its memory. Who will remind Hiroshima that Hiroshima was there? […] There’s nothing in the museum of the crime that points to the name of the killer: ‘The plane came this way, from a base in the Pacific.’ Is this collusion, or is it kowtowing? As for the victims, they need no names. […] Inscriptions on the wall explain degrees of death—from burns, smoke, poison, or radiation. Preliminary exercises for a more comprehensive global killing. Preparing for the end. Nowadays, the Hiroshima bomb’s destructive power makes it seem primitive nuclear weaponry. Yet, it has enabled scientific imagination to write the scenario of the end of the world: an enormous explosion, a gigantic explosion that will resemble the initial formation of the globe with its organised chaos of mountains, wadis, plains, deserts, rivers, seas, slopes, lakes, wrinkles, rocks, all the beautiful variety of an earth glorified in poetic praises and religious ceremonies (Darwish 2013, 84–85).

The excerpt above is from Mahmoud Darwish’s *Memory for Forgetfulness*, a text of extended prose poetry reflecting on the bombardment of Beirut by Israeli forces in 1982. Darwish (1941–2008) was considered the national poet of Palestine but is well-known around the Arab world and internationally. In *Memory for Forgetfulness*, Darwish commemorates Hiroshima Day—6 August—in 1982, drawing parallels between the two warzones, Beirut and Hiroshima, and the experience of being in a city undergoing near-apocalyptic destruction. Darwish situates the violence Beirut was undergoing within a broader historical framework, as opposed to depicting it as an isolated incident (Goldberg and Moore 2013, 33). By ‘remembering’ Hiroshima, despite the ‘American attempt to make it forget its name’, he identifies the use of the atomic bomb as a starting point for new forms of industrialised warfare, for imaginations of the end of the world, politicising its memorialisation (Neiman 2015; Zolberg 1998).

Darwish highlights the white-washed legacy of Hiroshima, referring both to the act of forgetting and the making of the city, after its destruction, into a symbol of peace (Lifton and Mitchell 1996; Zwigenberg 2016). Even today, the Hiroshima Peace Memorial Museum uses the passive voice to narrate history: ‘A single atomic bomb indiscriminately killed tens of thousands of people, profoundly disrupting and altering the lives of the survivors’ (2023). The removal of the actor depicts the bomb as having agency, or as a natural disaster. Darwish’s text is part of a larger body of work considering intersections between the atomic bombings of Japan and more recent cases of warfare, as well as the continued nuclearisation of the Earth (Deutsche 2011; Masco 2014; Olesen 2020; Shapiro 2015; Taylor and Jacobs 2018).

The text can simultaneously be read as a history of Arab engagement with the nuclear condition. Darwish was not alone in his attempt to revive Hiroshima as a starting point for modern militarism. Syrian poet Wafiq Khansa’s (2001) prose *Qasidat Hiroshima* (*Ash August: The Hiroshima Poem*), depicts Hiroshima as a haunting birthplace for new modes of killing—a place that has been ‘exiled’ from imagination (2001, 10; 19; 23). Like Darwish, he links its unprecedented destruction with continued militarism and displacement. For both authors, Hiroshima represents a beginning—serving as a mythical etymology for subsequent violence in the international system. ‘In Hiroshima, all death-roads meet,’ Khansa writes, ‘And they crisscross in Nagasaki, the two houses of doom, of acid rain, of nuclear dust, the [two] houses of human catastrophe’ (2001, 14–15).

Using these reflections as a point of departure, this article analyses stories of the atomic age from the Arab world. I focus on how people imagined ‘the atom’ in the immediate aftermath of the bombings of Japan, particularly how these imaginations crossed the boundaries of the real and fictional, which were increasingly blurred by the atomic age. Hysteria, speculation, exaggeration, and even mysticism shaped how people encountered and made sense of the nuclear age before doctrines such as deterrence emerged; yet, these more affective archives of world politics are often overlooked in International Relations (Bleiker 2012; Callahan 2020). To better understand the nuclear condition, I suggest, we need to consider these everyday, affective and visceral experiences of nuclearisation. Such stories illustrate how the atomic bomb suddenly entered people’s lives, becoming a new reference point, while shaping and structuring political possibilities (Jasanoff and Kim 2009; Krige and Wang 2015).

In addition to providing empirical examples of nuclear thought from the Arab world, this article makes several theoretical and methodological contributions. Considering the nuclear age from below, it demonstrates how popular imaginaries can elaborate forgotten, and obscured, nuclear experiences; as such, it challenges state-centric perspectives on nuclear politics and the invisibility of regions like the Middle East from discussions on the nuclear condition. By focusing on ‘semi-fiction’, the article analyses the intertwinement between fact and fiction, between history and the future, between imaginations and political alternatives (Babík 2019; Hom 2018; Pelopidas 2022; Sparks, Brincat, and Aistrope 2022). I perceive reality and fiction as not only deeply intertwined but also co-constitutive. As Considine (2022a, 2022b) demonstrates, our understanding of the nuclear age continues to rest on, and mobilise, a powerful ‘origin story’ of scientific brilliance. In tracing stories and mythologies surrounding the bomb, I pay special attention to its intertwinement with everyday life.

The next section expounds on affective archives and the genre of semi-fiction. The empirical sections discuss stories of the atom: (i) the bomb’s encroachment into mundane lives; (ii) speculative reports and pseudo-scientific predictions; and (iii) superstitions and rumours imbuing the bomb with magical undertones. Together, these sources indicate how people were thinking about, imagining and processing the atomic age. The conclusion reflects on the implications of this study.

## Affective archives, storytelling and semi-fictional historical sources

Nuclear weapons in the Middle East have been studied predominantly through fears of horizontal proliferation (Therme, Egeland, and Taha 2022). These fears tend to be rooted in nuclear Orientalism, the sense that nuclear weapons are particularly dangerous in the hands of states in the Global South, deemed uniquely irresponsible, irrational or ideological (Gusterson 1999). This research posits affective archives as a way to problematise and disrupt the conventional literature on the nuclear revolution in IR, which upholds assumptions of nuclear desire and deterrence (Biswas 2014). Despite its abstract nature, nuclear deterrence has been treated as a truth, a fact of world politics backed by historical evidence. Critics invoke empirical data—an alternative reading of the lessons of history—to suggest that deterrence is ineffective, risky or fallible (Lebow and Stein 1995; Sagan 1993; Wilson 2008). But deterrence is also a powerful story within the nuclear order, enmeshed with material interests (Egeland 2021; Egeland and Pelopidas 2022). Similarly, the US’s bombings of Japan have been explained using the Stimson narrative, a retroactive justification claiming the bombs saved lives; while debunked extensively, this account lives on as myth (Bernstein 1999; Miles 1985; Pelopidas and Egeland 2020).

Meanwhile, Arab thought on nuclear weapons has been studied through the perspectives of sovereign states, rather than ordinary people and colonised communities. Through its engagement with the blurred lines between real and fictional, this article aims to centre everyday people, working more actively towards a nuclear history from below (Hecht 2012; Van Munster 2021; Williams 2011), that is, a nuclear history that revolves around people’s everyday experiences, rather than the perspectives of official actors. Scholars have previously sought to develop nuclear histories from below by looking at experiences of uranium workers, resident families in nuclear communities, even scientists in laboratories (Brown 2013; Gusterson 1996; Hirschberg 2022; Voyles 2015), as well as embracing aesthetic and affective approaches (Choi 2023; Lauzon and O’Brian 2020; Särmä 2019). The objective is not only to add supplemental viewpoints and data points, but also through alternative interpretations of nuclear weapons and politics, to destabilise hegemonic historiography. For example, ambiguity and confusion tend to disappear from many accounts of the nuclear age, particularly national histories. However, as I will show, these sentiments characterised people’s early experiences of atomic weapons.

The article consults sources dealing with the period between August 1945 and 1948. This period is chosen precisely because it was a time of much speculation on the atomic age, captivating the imaginations of authors, journalists and observers, enabling an in-depth focus on the stories and sentiments that accompanied the atomic age. The year 1948 is chosen as the end range, as it marks the first Arab-Israeli war and the ensuing Palestinian Nakba, resulting in major political transformations. At the same time, in 1949, the emergence and later popularity of the transnational anti-nuclear Peace Partisans movement subsumed debates on, and ultimately resistance to, nuclear weapons in the Middle East (Wittner 2009, 25-28).

The sources selected for inclusion are eclectic, deliberately, to show that these sentiments and stories were not unique to one form of expression but permeated different genres. The sources straddle the two supposedly distinct realms of real and fictional: they include a memoir, popular science book and news and magazine articles speculating on the technological, political and mystical aspects of nuclear weapons. I therefore opt to describe the sources as semi-fictional. They share a key premise: they were not intended to be consumed as fiction. Even if they did not represent objective reality, they were produced as historical or scientific facts about the nuclear age, despite being fundamentally speculative. The intention of these sources was not to obscure; they attempted to understand the nuclear age amid regimes of ‘non-knowledge’ (Aradau 2017), namely uncertainty, secrecy and ignorance—often deliberate outcomes of securitisation (Masco 2002; Wellerstein 2021).

These sources drew upon fantasy to reflect on the nuclear age; these semi-fictions became part of people’s lived realities of the nuclear age. The term ‘semi-fiction’ has been used by literary and film scholars to describe writing that lies between fiction and non-fiction, stories stuck between memory and forgetting or imagining, which can be both historical and conjectural (George 1995). For IR scholars, semi-fiction is not only relevant to nuclear issues but can be useful to illustrate how world politics is shaped by perceptions and imaginations as much as reality (Sylvester 2015).

Despite their inaccuracy, semi-fictional stories of world politics can play a role in mediating uncertainty. Even today, the extent of nuclear destruction is unknown and unknowable (Pelopidas 2020), despite illusions of controllability, which overlook factors such as luck (Pelopidas 2017). Semi-fiction is highly pertinent to the study of nuclear weapons, which themselves pushed the limits of human imagination. Indeed, humanity seems unable to consider the destructive consequences of its innovations, a concept described by Günther Anders as a ‘Promethean discrepancy’ (Anders 1962; Van Munster and Sylvest 2019). Nuclear weapons were not only unknown, especially in this early period, but their destruction was so immense it seemed unfathomable. As I will show, the fact that a great deal was still undetermined drove people’s desire to know, understand and index ‘the atom’ and its impact, although rather than demystify the nuclear age, they paradoxically reinforced its sense of mystery.

By describing this genre as semi-fiction, I do not intend to reify a universal historical reality or suggest that fiction is distinct or disconnected from history. Instead, I aim to open avenues within IR for blurring the boundaries between the supposedly distinct categories of fact and fiction, for understanding history subjectively, studying a moment in time through the thoughts and experiences of agents. As John Caughey (1984) demonstrates, people inhabit multiple, interrelated social worlds, including imaginative ones, constructed through cultural inventories and repertoires that inform interpretations of political events, which often muddle the gap between the ‘two worlds of the actual and the fictive or vicarious’ (Malmsheimer 1986, 38). While memoirs are often dismissed for being neither accurate nor literary, it is precisely this undefined space that makes them valuable (Rak 2013). Still, like all sources, semi-fictional ones are also undoubtedly structured by power dynamics, as the perspectives described in this work are ones that were recorded and/or heard, reflecting a degree of privilege.

The methodological scope of this research is linguistic, rather than national. It works to transcend the methodological nationalism characterising nuclear studies, seen as part national histories and security (Abraham 2006). This national orientation prevents us from making useful connections about the global logics of colonialism and capitalism (Axster et al. 2021) and from conceptualising non-national spaces (Adamson 2016). Intellectual and scientific conversations in Arabic-language publications traversed national boundaries, and individuals travelled, worked and had personal and professional connections in different countries. By August 1945, the demand for decolonisation had engulfed the Arab world: popular and assertive nationalist movements had created a common reference point. Even as communities underwent unique national struggles, they considered how regional and global developments would affect decolonisation. As such, nuclear imaginaries in the Middle East were not insular; they were written by individuals who consumed and translated international news and took part in transnational conversations about science, decolonisation and the future.

## Hysteria, eggs and tennis balls

A memoir written by an Egyptian-born British medical professional, Ellis Douek, comically underlines the chaos associated with reporting on the atomic bomb. The author was born in Cairo in the 1930s to a wealthy Jewish family with Iraqi and Syrian roots, and the memoir chronicles his childhood and adolescence in Egypt, his studies in Europe, and ultimately, his exile from Egypt to Europe, where he settled in the UK and became a celebrated surgeon.^1^ Nuclear weapons and politics are not the main focus of the memoir, but the bombing of Hiroshima is discussed in passing as the author shares stories from his formative years in Egypt. Unlike other sources included in this study, Douek’s is a retrospective account of the events—and the only source in English. Yet, Douek’s hyphenated identity and expulsion from Egypt should not be treated as an indication of a lack of credibility and authenticity; rather, these factors are reasons to include his perspective, especially given the profound cultural role played by Jews in the Middle East at the time (Hammad 2022; Somekh 1988; Silver 2022; Bashkin 2012; Olsen 2021). Douek’s Egypt was multi-religious and multinational, and while the family had connections with colonial powers, it is evident throughout in the memoir that he viewed himself as Egyptian, even long after his exile.

The anecdote dealing with the atomic bomb reflects the memoir’s broader mission to demonstrate worldliness. The author and his family were attuned to global events, with rapid political and technological changes seen as having a direct impact on his life. Douek only dedicates a couple of pages to his memories of the atomic bomb, yet these pages demonstrate the extent to which the unveiling of the atomic bomb triggered emotional responses. The fact that Douek includes these depictions—largely forgotten today—suggests that they had a lasting influence. The memoir shows how the introduction of the bomb as a technological phenomenon and a military object was confusing, prone to embellishment. This is all the more vivid given that the author was a child, still learning about the world.

Douek recalls that on 6 August 1945, there was a sudden appearance of newspaper vendors to the upscale beach area in Alexandria where his family was on holiday (2004, 117–118). The floating papers he describes illustrate the importance given to the atomic bombing, which disrupted everyday life even in parts of the world that were geographically distant from both the United States and Japan. Through the metaphor of this intrusion, we recognise the atomic bombing as a global event (Gordin and Ikenberry 2020; Hogan 1996; Rotter 2008; Sherwin 2003; Zwigenberg 2014). Douek recounts his initial curiosity, as he swims back to the shore to understand the meaning of this interruption, as well as his sceptical reaction to his aunt who hurriedly spilled the news of the bomb. As she was generally ‘driven by drama and hysteria’, per his description, ‘She could not resist being the first to tell us of this tiny bomb, the size of an egg it was said, which had vaporised a whole city’ (2004, 118).

The young Douek was understandably suspicious of this story. It was only after receiving the news from a more reputable source, his parents, who confirmed that the atomic bomb was indeed small, that he accepted the story as credible and not merely an exaggeration, or even a complete fabrication, by his aunt. Douek provides a scientific rationale for the incorrect visualisation of the bomb as an egg, suggesting that the uranium used in the atomic bomb was around the volume of an egg. ‘I later studied the diagrams in the newspapers for myself’, he writes (2004, 118), suggesting the strangeness of the atomic bomb continued to intrigue him and that he would dispel the myths circulating through scientific explanations. Describing the bomb as being the size of an egg is in line with much of the official—and erroneous—reporting in a variety of Arabic-language media (Taha 2022a).

The frenzy about the atomic bomb was a common response. Confusion and ‘dramatisation’ were not limited to his aunt, as imaginations ran amok. Douek recalls another absurd incident, in which his older cousin was apprehended by police in Cairo on his way back from a tennis club. The cousin was reportedly handcuffed and taken to a police station. Like everyone else, a policeman had heard the shocking reports of the atomic bomb and discussed its ‘small’ size with his friends; as a result, he mistook the cousin’s tennis balls for atomic bombs (2004, 119). Tennis, a sport associated with wealth and played in exclusive and private spaces was a luxury mostly enjoyed by the elite, and therefore would not have been accessible to policemen, who came from poorer backgrounds. The story ends with the policeman apologising for his mistake and the cousin tipping him as a gesture of goodwill.

Even if the story is a parody—and a reflection, or embrace, of class distinctions and segregations within Cairo—it illustrates two key points. First, the emergence of the atomic age was being widely discussed, by everyone, across different class and religious lines. It was the talk of the day. It was experienced and imagined differently depending on one’s circumstances, yet people throughout the country were engaged in speculative conversations about its meanings and potential transformative effects. Second, during these conversations, the details of the bomb were grossly misunderstood and therefore subject to confusion—whether by the children, the aunt, the parents or the policeman. These characters are caricatures of their social roles: the naïve yet curious child; the gossiping and hysterical aunt, who simply cannot control herself; the knowledgeable, trust-worthy and wise parents; the wealthy and sporty but polite tennis player; and finally, the illiterate policeman who feels threatened by things he does not know or fully understand, particularly things that are ‘foreign’*—*in this case, both the sport of tennis and the atomic bomb.

The memoir reflects confusion over the nuclear condition and the ways in which atomic bombs were suddenly, bizarrely even, part of everyday life. As people unexpectedly encountered the phenomenon of the atomic bomb, they projected it through everyday objects. As an unknown and undefined object, the bomb could be transposed onto anything, including mundane items, such as an egg or tennis ball. Depicting the bomb in a tennis ball or an egg can be seen as a reflection of society’s strong emotional reaction, whether fear, excitement or intrigue. While it was a distant object with an unfathomable destructive power, the atomic bomb seems strikingly close–almost reflecting an intrusion into people’s lives.

Both representations reflect the dominant sense of awe that surrounded discussions of the bomb. In a context where we are, as Anders wrote, ‘unable to visualise what we actually producing’ (1962, 496), the bomb can be represented through these everyday objects. In other words, its reputation as a small, highly destructive object meant that its actual size was made irrelevant in these imaginations. Indeed, there is a sharp contrast between nuclear weapons—objects that are extraordinary, complex, and hyper-scientific—and the way they are rendered through these everyday objects. As I will show below, although nuclear weapons became intertwined with everyday objects and lives, they were still dealt with as magical or mythical.

## Speculation and pseudoscience

Early reports on the atomic bomb in the press were arguably as inaccurate and unreliable as Douek’s aunt. The contingency of early interpretations was not restricted to areas far-removed from the bomb; as Michael Gordin (2007) shows, even the US military did not fully anticipate or comprehend the implications of the atomic bomb. In Japan, news reports relayed inaccurate information, declaring, for example, that the atomic bomb had been parachuted into Hiroshima (UPI 1945). The confusion associated with the initial reporting on the bomb is unsurprising, given strict US censorship on the effects of the bombings and control by scientists and experts over information pertaining to the topic of radiation (Brau 1991; Brodie 2015; Lindee 1994; Low 2020).

There was a seemingly boundless space to interpret the atomic age. Globally, the atomic age created utopian and dystopian ideas (Boyer 1985; Masco 2015; Zwigenberg 2022), but these were especially pronounced among people amid decolonisation struggles (Abraham 1998). As a result, speculation and pseudoscience became intertwined with dreams of political and technological independence. In Arabic news reports, there was no clear line between fact and fiction in terms of the effects of the atomic bomb—which were described as mysterious—and its size and shape—imagined to be tiny, as suggested above—or its implications for science and technology, opening vast new horizons for humanity, as well as global politics and security. The meaning of this new weapon was undetermined: all that was known was that it was a small object with overwhelming destructive capabilities.

After the revelation of the bomb, reporting was characterised by a sense of optimism, represented through enthusiasm for the potential of atomic energy. This positive spin was deliberately emphasised by US President Harry Truman, whose statement announcing the use of the atomic bomb in Hiroshima mentioned energy, science and the ‘understanding of nature’s forces’ (Miller Center 2023)—itself a speculative statement. This sense of ‘atomic schizophrenia’ (Kaur, 2013) regarding the reception of the attacks in Japan was widespread in the Middle East and elsewhere. Journalists, scientists and intellectuals regularly invoked the potential peaceful applications of atomic technology, yet they did so alongside coverage of its destruction (Taha 2022b). They sensed that the bomb was an extreme—it was the pinnacle of both destruction and human brilliance. For instance, a story in the largely pro-British Egyptian newspaper *Al-Muqattam* described the atomic bomb as ‘the most destructive tools of destruction and the most powerful powers of nature’ (1945, 1). This eagerness was evident in reports on atomic technology more broadly: for example, on 10 August 1945, the Damascus-based daily newspaper *Alif Baa*, associated with the nationalist movement against the French mandate in Syria, was already referring to the potential uses of atomic energy in peacetime. In its report on how atomic energy constituted a new juncture, the newspaper alleged that ‘within 100 years, we will be able to travel to the moon using atomic energy’ (1). The report referred vaguely to British scientific sources, although it does not at any point clarify why 100 years specifically are needed, or how this number—so far into the future—was reached.

Not only was the information unverified, but in many cases, it contained dimensions akin to fantasy or science fiction. Like *Alif Baa, Falastin,* the most prominent Arabic-language newspaper in mandatory Palestine, reported the claim that atomic technology would enable humans to reach the moon. It is, however, worth emphasising that such confusion, and perhaps even confidence, in atomic technology did not necessarily limit the scope of critique. The headline of *Falastin* stated: ‘The first atomic bomb (erases) the Japanese [city of] Hiroshima and allows people to reach the moon’ (1945, 1). This is a clear example of the speculation characterising news reports, but it simultaneously highlights how the discussion of the bomb’s material and destructive capacity was linked to a reflection on its scientific potential. The headline is characteristic of this duality, combining a recognition of the scale of destruction in Japan with a degree of scientific enthusiasm for atomic technology and its potential to propel humanity to new places. In other words, the fuzzy distinction between fact and fiction did not necessarily undermine critiques of the nuclearisation of the world.

Another Palestinian newspaper, the Haifa-based weekly *Al-Ittihad* (affiliated with the communist party) emphasised the bomb’s potential to propel humanity towards new horizons, and it included a range of facts and figures about the bomb. Published on 12 August, the article contained strangely precise—and shockingly incorrect—details. The data included the size of a city that the bomb is capable of destroying (2 million people), how long 1 kg of atomic energy could power a motor (250 years—with no interruption), how much power 1 kg of atomic energy possesses (equivalent to 25 million kilowatts and 660 thousand gallons of fuel), and the size of the atomic bomb itself (which does not exceed the size of an egg) (1945a, 1). The article is written with a sense of scientific authority, and these figures suggest a technical expertise mediating this information. The tone of the article was enthusiastic regarding atomic science, but this is disconnected from discussions on ending the Second World War. In fact, another article on the same page in the same newspaper, directly below, declared, ‘The war on Japan will end in the coming days: the attack by the Red Army on Japan is progressing rapidly’ (Al-Ittihad, 1945b, 1), and contained no mention of the atomic bomb as having sped up the end of the war, in contrast to the dominant Western narrative of the bombing (Herken 1982). The imminent end of the war, rather, was attributed to the Soviet invasion of Manchuria. The use of the atomic bomb is divorced from the end of the war: although both events were considered worthy of the front page, they are treated as two separate events, warranting two different stories and news coverage.

Similar ideas were printed in *Al-Muntada,* a Palestinian magazine that explicitly promoted modernisation and paid special attention to science and technology. In its September 1945 issue, the magazine included commentary on what is known so far about the atomic bomb, which expressed similar, seemingly scientific, discussions. Describing the ‘miracles’ of the atom, the article notes that explosive materials the size of an egg can transform a large and bustling city into a cloud of dust. But it also describes how one ounce of fuel can move a 100-horsepower machine for 64 years continuously, day and night, and how one ounce of matter extracted from the atom is equivalent to the force generated by the Niagara Falls for eight months (1945, 5). Some of this information was clearly extracted from US-based sources already enthusiastic about the atomic age, while others were markedly regional expressions of the nuclear age, such as the reference to the egg.

Speculation was not unique to one political orientation. As we can see in the examples laid out above, it traversed the political spectrum—ranging from venues focused on modernist ideas to nationally oriented projects to communist movements to those aligned with colonial powers. Spaces that were dedicated to, and enthusiastic about, science and technology were just as likely to embrace fantastical ideas. The dependence on these precise figures to understand the power of the atom—to understand its capacity for destruction, but also for producing energy—suggests that it was still a technological fantasy. That it was yet unknown drove the thirst to understand, detail and quantify its impact and potential.

## Mysticism, mystery and magic

The atomic bomb was also discussed through the realms of the mystical and the mysterious. For example, in an op-ed on 12 August 1945 in the most widely circulating Egyptian newspaper *Al-Ahram*, statesman Abd al-Wahab ‘Azzam wrote about the ‘power of the atom‘, linking it to the work of Persian Sufi poet Farid al-Din al-Attar (1142–1220). ‘Azzam describes the bomb as an impressive reflection of ‘the human brain that can unearth the secrets of nature and recognise its power’, though he cautions that humanity is at the same time deriding this power through its use for the purposes of damage and destruction (1945, 3). The reference to ‘unearthing the secrets of nature’ implies that there is more to be learned about atomic science and that it contains unknown, and potentially even spiritual, dimensions—though still recognising its militarist dimensions.

Similarly, in a popular science book published in 1946, which purported to dispel some of the confusion around atomic science, Palestinian thinker ‘Ali Rashid Sha‘ath refers to black magic, witchcraft, and superstition—subjects sometimes invoked in discussions on exposure to radiation. Still not understood, radiation was seen as a ‘mysterious’ effect of atomic weapons (see, for example, *Al-Ahram* 1945a, 1; 1945b, 1). The city of Hiroshima, Sha‘ath writes, had been overshadowed by ‘strange magic’. He begins the book by recalling well-known myths and legends in the Middle East before declaring, ‘What we thought of as creations of the imagination and stories of superstition have now become an ordinary and unusual reality. Today we live in an age filled with creativity, magic, and wonders’ (1946, 6). The author does not deny the violence of the bomb but imagines it within the context of a new and potentially magical future—an extraordinary force that will shape the world (Taha and Olsen, forthcoming). Here, we see an intersection between modernity and science, on the one hand, encapsulated through the atomic bomb, and the occult and mystical, on the other, represented through magic, myths, and superstitions (Saler 2006). Like in Darwish and Douek’s reflections, Nagasaki is curiously absent—an indication that authors took Hiroshima to symbolise the nuclear condition.

Rumours about nuclear weapons continued to circulate in subsequent years. For example, reports in Israel mention that Palestinians believed that Jewish militias had—and were using—an atomic bomb in Safed in the north of Palestine in May 1948. Jewish militias had reportedly developed a mortar known as the Davidka that was inaccurate, yet extremely loud, which, as far as the myth goes, was mistaken for an atomic bomb (Morris 2004, 224; Rabinovich 2022, 108; *New York Times* 1969, 44; see also Abbasi 2004). Some sources suggest this rumour was deliberately and tactically spread by the Jewish militias operating in that area (Bar 2020, 341; Palumbo 1989, 113).

External factors encouraged this rumour. It was not only the sound and flash of the mortar, according to certain retellings of the story, but also an untimely rain that led to such speculation (Morris 2004, 224; Syrkin 1966). This impression may have been the result of reports that described the ability of nuclear weapons to change weather patterns—through discussions about the phenomenon of ‘black rain’ that engulfed Hiroshima—and to defy nature. One journalist describes Palestinian prisoners of war who believed Zionist militias had a ‘secret weapon […] which makes fire spout out of the earth and houses cave in without visible cause’ (Koestler 1949, 215).

In some ways, the story is a colonial fantasy. It reflects a recurring Israeli discourse on how Palestinians simply ‘fled’ during the war, a discourse that overlooks the violence inflicted upon Palestinians. These reports rehearse well-known Orientalist tropes about Palestinians and Arabs in general, being naïve and unintelligent, but also emotional, frantic and prone to frenzied behaviour. ‘They ran like mad and left the town completely’, reported a Yiddish newspaper’ (Der Tog 1949, 9). Here, the fear and confusion accompanying the atomic age were seen as a unique attribute to this community, rather than as a fundamental feature of the nuclear age more broadly, as shown above.

The story of the Davidka is memorialised in a public square in Safed, almost as a form of divine intervention. The monument notes the ‘miracle’ of this weapon that scared and displaced Palestinian residents, paving the way for Israeli control of the city, but it does not mention rumours about nuclear weapons. Yet, stories like this one can encourage us to rethink the lines between expulsion and forced evacuations and to pay closer attention to people’s experiences of the Nakba, as Nur Masalha reminds us (1988). In addition to being a colonial fantasy, though, the report reflects another fantasy as well: a dream of the myth of nuclear deterrence. Mere rumours of nuclear weapons, it suggests, are enough to ‘deter’ the enemy. And in fact, this fantasy would later become the cornerstone of Israeli policy on its nuclear weapons, which are officially governed by the principle of *amimut*, which translates into opacity or ambiguity, i.e., that Israel does not confirm or deny having nuclear weapons.

Until today, nuclear weapons continue to be intertwined with secrecy and mystery. Rumours abound in Palestine regarding the burial of nuclear waste in the West Bank (Stamatopoulou-Robbins 2020, 127–128), though Palestinian scientists have faced limitations in their ability to research nuclear waste, including Israeli restrictions on access and imports (Abu Arqoub 2015) Around 70 years later, many still speculate about the mysterious deaths of early Arab nuclear scientists, such as Samira Moussa, the first Egyptian female nuclear physicist who died in an accident during a brief visit to the United States in 1952 (El-Geressi 2019; Ghoneim 2011). Such incidents are depicted as precursors for subsequent well-documented assassinations of Middle Eastern nuclear scientists by Israel (Peck 2012), and although there is no evidence of a link between these events, we can see lasting the political impact of rumours and myths (see Aistrope and Bleiker 2018).

## Conclusion

Stories of world politics enable IR scholars to further unsettle and demystify the authoritativeness that has been attached to certain historical records. Challenging the fetishisation and alleged indexical quality of state and official archives, this article calls to also engage everyday material, speaking to the growing IR literature on aesthetics, affect and visualisation. Through these stories of the nuclear age, this research disrupts the prisms through which the nuclear age has typically been articulated—including ideas of deterrence, fears of proliferation, assumptions of desire. The nuclear condition cannot be understood only by looking at states; if we broaden the scope and consider alternative archives, we can listen to and tell different stories about the nuclear age—particularly its destabilising effect.

Semi-fictional sources reveal the extent of the Promethean discrepancy. As Anders wrote, ‘The apocalyptic danger is all the more menacing because we are unable to picture the immensity of such a catastrophe’ (1962, 496). It is not surprising that Douek, as a child, suspected the story of the atomic bomb to be a mere exaggeration. The nuclear age opens up a window into an unusually sudden change; after all, violence that was not possible, was mythical, or even unthinkable, quickly became not only possible, but also undeniably real. The semi-fictional sources described attest to this sense of rupture. People had to grasp to find ways to deal with and understand this disturbing transformation. Generally, the discussions laid out above point to this sense of incredulousness, yet combined with astonishment. How could humanity reckon with its ability to cause this much destruction?

However, Darwish was correct when he pointed out that new generations of nuclear weapons have become significantly more destructive, and that the Hiroshima bomb would now be considered rudimentary in comparison to the arsenals of nuclear weapons states today, which have been normalised as part of the Earth’s future (Pelopidas 2021), reproduced as common-sensical (Ritchie and Egeland 2018), and are even legitimised as necessary for global security (Tannenwald 2018).

The element of hyperbole is still apt. Like Darwish also notes, nuclear weapons reflect modern science’s engineering of apocalyptic scenarios and their appropriation from the realm of the divine. In her essay about Indian nuclearisation, Arundhati Roy eloquently sums up the nuclear condition:

If you are religious, then remember that this bomb is Man’s challenge to God. It’s worded quite simply: *We have the power to destroy everything that You have created*. If you’re not religious, then look at this this way. This world of ours is four billion, six hundred million years old. It could end in an afternoon (italics in original, 2016, 64).

In the context of this Promethean discrepancy, people have relied on a range of emotions—from fear to enthusiasm—and mobilised hypermodern scientific projections alongside mythological and mystical implications. As such, IR scholars need to consider the overlap between ‘real’ and ‘fictional’ and to embrace the muddling of such distinctions.

Finally, this article insists that the nuclear condition must be understood affectively—that self-proclaimed rational theories such as deterrence fall short of explaining the impact of the nuclear age on communities and individuals. While the stories described above demonstrate that people were profoundly affected by the emergence of nuclear weapons, they did not immediately gravitate towards building their own weapons, and they did not link nuclear weapons with security. Through these accounts, we can undo the very assumption of nuclear weapons as stabilising. We need to more actively rethink established ‘origin stories’ while considering future-oriented imaginaries that may have been obscured. In doing so, we can challenge the continued reproduction and normalisation of the nuclear-armed world as inevitable (Pelopidas 2021). In this vein, this work aims to reinterpret the relationships between past and future (Andersson 2018; Mallard 2018), beyond a positivist return to history to reconsider the lessons of the past (Vaughan-Williams 2005).

While numerous scholars have emphasised the importance of fiction for understanding world politics, this article takes on the task of looking at materials that were not meant to be consumed as fiction—highlighting that nuclear technology straddled fiction and non-fiction, especially in this early period. The analysis of these semi-fictional historical archives and texts and their often-absurd elements illustrates how nuclear imaginations circulated globally. These sources can fill in historical gaps, highlighting voices and perspectives that have been neglected, yet without imposing one universal truth upon history. This approach can, as Lemke et al. (2023, 10) remind us, open up historical IR to new avenues, focusing not only on facts, such as what happened and why, an approach which often prioritises voices of powerful actors, but also imaginations and alternatives that centre the agency of different communities.

Beyond its specific case study, this article emphasises the importance of alternative histories of the nuclear age, which centre people’s experiences. These allow us to insert actors typically seen as ‘non-nuclear’ as relevant thinkers, not only passive observers. We can accordingly understand sentiments that were subsequently translated into policy action, whether through transnational movements like the Peace Partisans, Third World networks such as the Bandung Conference, or contemporary movements for nuclear abolition, such as the Treaty for the Prohibition of Nuclear Weapons (Intondi 2019). Driven by people’s experiences and sentiments, these forums have resisted the concepts and frameworks of the nuclear literature that continue to dominate the vocabulary of policymakers and academics, such as deterrence and proliferation. Further research would benefit from excavating everyday nuclear thought and its potential for political transformation.

## Footnotes

1 On the expulsion of Jews from Egypt in 1956, see Beinin (1998). Douek wrote another memoir in 2022, *To Hear Again, To Sing Again*, which focuses on his experience as a pioneer of hearing implants.
